# Women’s Perceptions and Knowledge Toward Episiotomy in Qassim Region, Saudi Arabia

**DOI:** 10.7759/cureus.55383

**Published:** 2024-03-02

**Authors:** Lubna A Alsuraykh, Aseel A Alnadawi, Aeshah Alharbi, Kadi A Alhumaidi, Nadiyah Alhabardi, Raghad Almarshud

**Affiliations:** 1 Medicine, Unaizah College of Medicine and Medical Sciences, Qassim University, Unaizah, SAU; 2 Obstetrics and Gynecology, Unaizah College of Medicine and Medical Sciences, Qassim University, Unaizah, SAU; 3 General Surgery, Unaizah College of Medicine and Medical Sciences, Qassim University, Unaizah, SAU

**Keywords:** saudi arabia, cross-sectional study, maternal outcomes, intrapartum decision-making, women’s health, episiotomy

## Abstract

Introduction: Episiotomy, despite being one of the most common interventions during childbirth, carries significant risks and uncertain benefits. Previous global studies highlight varying awareness levels and practices, with decreasing episiotomy rates attributed to increased knowledge. This study aims to assess women’s knowledge to enhance intrapartum decision-making and communication between patients and obstetricians, ultimately improving maternal outcomes in the region.

Methodology: The study was a cross-section design. It was conducted through an online survey that was distributed by different social media platforms (Twitter, WhatsApp, and Telegram) from February 2023 to January 2024. It included women living in the Qassim region, Saudi Arabia, who were 15 years old or older. Data was analyzed using the SPSS program (IBM, Chicago, Illinois, USA).

Results: Among the 402 participants, 62.7% demonstrated awareness of episiotomy, with 94.0% accurately identifying it as a surgical cutting with scissors. About 82.5% acknowledged that not all women require episiotomies, while 48.8% recognized the necessity of anesthesia before the procedure. Understanding the indications for episiotomy varied, with facilitating and accelerating childbirth (64.3%) and dealing with a large baby (62.3%) being the most recognized reasons. Impressively, 90.5% believed that there are methods to avoid perineal cutting, with knowing the correct mechanism for pushing during childbirth (69.4%) and exercise (54.4%) being the most acknowledged preventive measures. Regarding post-cutting care, antibiotics (61.5%) were identified as essential, followed by analgesia (52.8%) and laxatives (48.8%).

Conclusion: The study reveals a notable awareness among participants, with a majority demonstrating a solid understanding of the procedure, its indications, and post-procedure care. It identified specific knowledge gaps, such as the need for anesthesia awareness and divergent beliefs about post-episiotomy care practices.

## Introduction

Episiotomy is an intended incision made through the perineal body to enlarge the vaginal orifice during the second stage of labor to ease the parturition. It is one of the most common interventions in the delivery room. A shorter second stage of labor and a decrease in the incidence of fetal skull fractures and cephalohematomas were said to have positive effects on the fetus.

The surgical incision of the perineum (episiotomy) was recommended for the prevention of maternal trauma and improvement of fetal outcomes as the medical community started to regard birth as a medical procedure conducted in a hospital rather than a physiologic process occurring at home [1]. Episiotomy incisions can be made in a variety of methods, including the midline, modified-median, mediolateral, J-shaped, lateral, anterior, and radical. Three of these are most frequently used. The medial episiotomy is a midline incision of the perineum down to near the anus. Mediolateral episiotomy incision is between 40 and 60 degrees in the left or right of the anal canal. Lateral episiotomy incision is at either 4-5 or 7-8 o’clock at an angle away from the midline of 40-60 degrees.

The studies demonstrated significant risks associated with episiotomy; they found a lot of complications such as perineal lacerations, hemorrhage and increased blood loss, wound site edema, wound site infection, anal sphincter and rectal mucosal damage, urethral injury, bladder injury, hematoma formation, pain, and dehiscence which consider short-term effects. On the other hand, the long-term effects are chronic infections, anorectal dysfunction, urinary incontinence, pelvic organ prolapse, sexual dysfunction, and pain [2].

There is no conclusive evidence for the effectiveness of the routine use of episiotomy, according to an evaluation of the studies about the risks and benefits of episiotomy. Many previous studies have shown a low level of knowledge and practice about episiotomy. In the United States, a research conducted by Frankman et al. [1], during the period between 1979 and 2004, discovered that the prevalence of episiotomies for all vaginal births dropped from 60.9% in 1979 to 24.5% in 2004. As liberal usage has been discouraged, the frequency of routine episiotomies has likely decreased, reflecting the drop in episiotomy usage [1]. Another study in São Paulo, Brazil, conducted by Wey et al. [3], during the period between April 2001 and April 2002 showed that 91.5% of 1,837 normal births involved episiotomies. A second survey conducted in 2006 showed a reduction in frequency, with that year's frequency being 44.7%. These rates are still far higher than the 10% that the WHO advises [3]. Also, in Canada, a research study conducted by Klein et al. [4] in 2011 concluded that many women did not fully understand the advantages and disadvantages of episiotomies [4]. In a research study conducted by Thompson et al. [5] in Australia, the results showed that 34% of women were not consulted about episiotomy. Also, 26% of the women who had previously undergone an episiotomy reported that they had neither been informed nor consulted [5]. In France, a study conducted by Reinbold et al. during the period between 2004 and 2009 demonstrated that the episiotomy rates dropped dramatically from 55.7% to 13.3% after the French Guidelines on Episiotomy were introduced [6].

Moreover, in Northwest Ethiopia, a research study conducted by Woretaw et al. [7] showed that the magnitude of episiotomy practice exceeds WHO recommendations. In a comparison of mothers whose birth spacing was less than two years and more than two years, episiotomy practice was 4.76 times more likely compared to mothers whose birth spacing was more than two years [7]. Also, in a research study conducted by Abubakar et al. over an eight-week period in Nigeria, there were a number of women interviewed, of which 65.5% had heard of episiotomy [8]. Additionally, in Turkey, a research study conducted by Karaçam et al. in 2013 found that the episiotomy rate was 56.3% [9]. Furthermore, in Iraq, a research study conducted by Muhammad found that the vast majority of primiparous women lack adequate knowledge and practice regarding episiotomy and perineal care [10].

Likewise, in Lebanon, a research study conducted by Kaddoura et al. [11] during the period between January 2009 and January 2014 observed that the rate of episiotomy was exceptionally high, with 97.4% of ladies getting an episiotomy in 2009. A significant reduction in the rate was identified from 97.4% in 2009 to 73.3% in 2014. The episiotomy rate stays higher than suggested by WHO [11]. In Oman, a research study conducted by Al-Ghammari et al. in 2016 found that the episiotomy rate was 66% [12]. Finally, in Saudi Arabia, two research studies were conducted, one in Buraidah in 2014 found an episiotomy rate of 51.20%. Another recent study conducted in Jeddah in 2015 revealed that the rate of episiotomy had increased from 35% in 2012 to 36.4% in 2015 and that we were still far from following the new American College of Obstetricians and Gynecologists guidelines to reduce the rate of episiotomies. The majority of women reported being aware of episiotomy; women with a history of episiotomy were six times more aware than women with no history of previous episiotomy [13,14].

Episiotomy has many meanings for various people and groups, with multiple meanings depending on the social setting, one's background in the workplace, and one's own experiences. Also, there is a lack of understanding and information about this practice. As a result of the lack of awareness, many expectant mothers in our setting have not accepted episiotomies. The purpose of this study is to determine the degree of women's knowledge about the episiotomy procedure in the Al-Qassim region, Saudi Arabia. A critical component of enhancing the standard of care for intrapartum decision-making is assessing patients' perceptions of specific procedures. Moreover, better patient and obstetrician communication will help to improve overall outcomes.

## Materials and methods

The study was conducted as an observational, cross-sectional study from February 2023 to January 2024, targeting women in the Qassim region through survey links via various social media platforms (Twitter, WhatsApp, and Telegram). The sampling technique was non-probability sampling, with inclusion criteria being all women living in the Qassim region who are 15 years old or older, while exclusion criteria were women living outside of the Qassim region and who are younger than 15 years old. Ethical approval (607-45-2220) was received from the Regional Ethical Committee of Qassim Region, the Kingdom of Saudi Arabia, and participants were ensured confidentiality and the freedom to withdraw from the study at any time.

The data was collected using a structured questionnaire consisting of three sections. The first section included sociodemographic factors. The second section included questions to assess the knowledge about knowledge and practice of the studied participants about episiotomy. The third section included questions of the previous experience of episiotomy of the participants.

The native language of the study authors was Arabic; therefore, the questionnaire was first prepared in Arabic and then translated into English. An English language professional was requested for the translation. The Arabic questionnaire was then translated into English. The English version was translated back into Arabic to verify that the English version contains the actual meaning. After the translation, a pilot study was conducted to validate the questionnaire. A total of 15 responses were collected, and a reliability analysis was performed. A high score from the analysis revealed that the questions included in the questionnaire were reliable, and no modification or exclusion of any question was required.

The required sample sizes were calculated using the following equation:

n = z12p (1p)/ d2

where n is the sample size, z=1.96, and p is based on 50% of the value. Both descriptive and inferential statistical analysis of the data was carried out. Simple descriptive statistics of the sociodemographic characteristics and other categorical variables in the form of frequencies and percentages were calculated and tabulated. For continuous variables, means/medians and standard deviation/interquartile ranges (IQRs) were reported as measures of central tendency and dispersion, respectively, owing to the relatively non-normal distribution of variables assessed by Kolmogorov-Smirnov test (p<0.001).

For the questions assessing the knowledge and awareness of episiotomy, a score of 1 was assigned for each correct response and these were summed up to calculate the total knowledge score of each participant. Thus, the total possible score of a participant ranged from 0 to 25. The scores were compared among participants of different sociodemographic characteristics. The comparison involved inferential statistical analysis namely the non-parametric Mann-Whitney U Test and the Kruskal-Wallis test. Significance was established at a p-value of 0.05 indicating a 95% confidence interval. All statistical calculations were performed using SPSS version 27.0 (IBM, Chicago, Illinois, USA). 

## Results

The study encompassed 402 participants from diverse sociodemographic backgrounds. The majority fell within the younger age brackets, with 44.8% (N=180) aged between 15 and 24 years and 23.1% (N=93) falling within the 25 to 34 age group. Saudi nationals constituted the overwhelming majority at 98.3%(N=395). Educational attainment was skewed toward higher education, with 84.6% having completed university or higher studies. Marital status varied, with 46.8%(N=188) married, 49.5% (N=199) single, and smaller percentages being divorced (2.5%)(N=10) or widowed (1.2%)(N=5). In terms of childbirth history, a notable proportion (54.0%) (N=217) reported no prior births, while 28.9% (N=116) had between one to four births and 17.2% (N=69) had five or more. Interestingly, a substantial portion (62.7%) (N=252) were aware of perineal cutting during childbirth, contrasting with 37.3% (N=150) who had not heard of this practice (Table 1).

**Table 1:** Sociodemographic Characteristics of the Participants The data has been represented as N, %.

		N	%
Age group	15-24	180	44.8%
	25-34	93	23.1%
	35-44	70	17.4%
	More than 45	59	14.7%
Nationality	Non-Saudi	7	1.7%
	Saudi	395	98.3%
Education	High school and below	62	15.4%
	University and above	340	84.6%
Marital status	Divorced	10	2.5%
	Married	188	46.8%
	Single	199	49.5%
	Widow	5	1.2%
Number of births	1-4	116	28.9%
	5 or more	69	17.2%
	None	217	54.0%
Have you ever heard of cutting the perineum during childbirth?	No	150	37.3%
	Yes	252	62.7%

Among the 252 participants who were aware of episiotomy (62.7% of the total 402 participants), a notable majority demonstrated familiarity with various aspects of the procedure. When asked to describe the procedure, 94.0% correctly identified it as a surgical cutting with scissors by a health practitioner. A significant portion (82.5%)(N=402) correctly answered that not all women require episiotomies during childbirth, while 48.8% recognized the necessity of anesthesia before the procedure. Understanding the indications for episiotomy varied, with facilitating and accelerating childbirth (64.3%) (N=402) and dealing with a large baby (62.3%) being the most recognized reasons. Impressively, 90.5% believed that there are methods to avoid perineal cutting, with knowing the correct mechanism for pushing during childbirth (69.4%) and exercise (54.4%) being the most acknowledged preventive measures. Regarding post-cutting care, antibiotics (61.5%) were identified as essential, followed by analgesia (52.8%) and laxatives (48.8%). An overwhelming majority (77.0%) acknowledged the importance of a doctor seeking consent before performing an episiotomy. Overall, the surveyed participants showcased a substantial understanding of episiotomy-related concepts, including its procedure, necessity, preventative measures, post-procedure care, and the significance of informed consent (Table 2).

**Table 2:** Participants’ Knowledge Regarding Episiotomy Correct answers are in bold. The data has been represented as N, %.

		N	%
Describe the procedure for an episiotomy (perineum = birth canal)	Surgical cutting with scissors by a health practitioner	237	94.0%
	I don't know	8	3.2%
	Tearing while pushing	7	2.8%
Do all women need an episiotomy during childbirth?	No	208	82.5%
	I don't know	33	13.1%
	Yes	11	4.4%
Is anesthesia required before cutting the perineum?	Yes	123	48.8%
	I don't know	66	26.2%
	No	63	25.0%
Indications of episiotomy	Facilitating and accelerating childbirth	162	64.3%
	A large baby	157	62.3%
	To avoid perineal tear	134	53.2%
	To assist in delivery	102	40.5%
	Fetal distress (weak fetal heartbeat)	52	20.6%
	Pregnant for the first time	42	16.7%
	Maternal fatigue from pushing during birth	41	16.3%
	Need to use obstetric forceps or vacuum.	39	15.5%
	Upon the patient’s request	38	15.1%
	Previous history of perineal tear	37	14.7%
	Routine procedure	28	11.1%
	Breech baby	25	9.9%
	Other	19	7.5%
	I don't know	2	0.8%
Do you think there are ways to avoid cutting the perineum?	Yes	228	90.5%
	No	24	9.5%
If yes, how it can be avoided	Knowing the correct mechanism for pushing during childbirth	175	69.4%
	Exercise	137	54.4%
	Pelvic muscle massage in the last weeks of pregnancy	134	53.2%
	Healthy eating	69	27.4%
	Other	5	2.0%
What do you think is important to take after cutting the perineum	Antibiotic	155	61.5%
	Analgesia	133	52.8%
	Laxative	123	48.8%
	Herbal	38	15.1%
	I don't know	31	12.3%
Do you think the doctor should take your consent before performing an episiotomy?	Yes	194	77.0%
	No	58	23.0%

In examining participants' knowledge regarding episiotomy wound care among 252 respondents, there emerged a nuanced understanding of post-procedure care. A substantial majority recognized the importance of hygiene practices, with 87.3% (N=402) acknowledging no harm in washing or showering daily after perineal cutting. Moreover, 97.2% (N=402) understood the necessity of frequently changing sanitary pads post-episiotomy for proper wound care. Participants (N=402) overwhelmingly agreed (98.8%) that keeping the wound clean and dry is vital in infection prevention. Additionally, 94.8% acknowledged the significance of avoiding constipation for optimal healing. Interestingly, there were contrasting opinions regarding specific practices, with 54.4% correctly disagreeing with the belief that washing the perineal area with salt aids wound healing and 43.7% believing that using herbs, like myrrh or aloe vera, aids sterilization and healing. Among those who believed in herb usage (56.3%), roughly half (54.9%) had used these herbs previously for the same purpose. Notably, there was an almost equal split regarding the use of only water for perineal washing, with 47.6% (N=402) advocating for it and 52.4% opposing it. This depiction of participants' perceptions highlights a nuanced understanding of various post-episiotomy wound care practices, showcasing both consensus and divergence in beliefs about specific care methodologies (Table 3).

**Table 3:** Participants’ Knowledge Regarding Episiotomy Wound Care The data has been represented as N, %.

	Correct Answer	Yes		No	
		N	%	N	%
Do you think there is no harm in washing or showering at least once a day after cutting the perineum?	Yes	220	87.3%	32	12.7%
Do you think you should change sanitary pads frequently after an episiotomy?	Yes	245	97.2%	7	2.8%
Do you think the wound should be kept clean and dry to avoid infections?	Yes	249	98.8%	3	1.2%
Do you think it is important to avoid constipation?	Yes	239	94.8%	13	5.2%
Do you think that washing the perineal area with salt can help the wound heal?	No	115	45.6%	137	54.4%
Do you think using some herbs (such as myrrh, aloe vera, etc.) helps sterilize and heal the wound?	Yes	142	56.3%	110	43.7%
If your answer is yes, have you used these herbs before for the same purpose? (N=142)	Yes	78	54.9%	64	45.1%
Do you think women should only use water to wash their perineum after an episiotomy?	Yes	120	47.6%	132	52.4%

Table 4 presents insights into the experiences of 252 participants regarding episiotomies during childbirth. Almost half of the respondents (48.0%) reported not having undergone an episiotomy, while 46.0% confirmed experiencing the procedure. Among those who had an episiotomy, a considerable majority (83.6%) (N=402) indicated that they were not consulted or given permission before the procedure. Regarding the time of suturing the wound post-cut, 92.2% reported it occurred within an hour. Notably, 44.8% (N=402) mentioned receiving anesthesia before the procedure. Reasons for undergoing episiotomy varied, with facilitating childbirth (56.0%) (N=402) being the most common, followed by routine procedure (33.6%) and assistance in delivery (31.9%). Complications post-episiotomy were reported by 62.1% of respondents, predominantly comprising perineal pain (33.6%), dyspareunia (26.7%), and suture disintegration (21.6%). This comprehensive depiction offers insights into the prevalence of episiotomies, the consent process, the timing of the procedure, the reasons behind the practice, and the spectrum of complications experienced by participants following episiotomies.

**Table 4:** Participants' Previous Experience of Episiotomy The data has been represented as N, %.

		N	%
Have you ever had an episiotomy during childbirth? (N=252)	No	121	48.0%
	Yes	116	46.0%
	I don't know	15	6.0%
Have you been consulted and given your permission? (N=116)	No	97	83.6%
	Yes	13	11.2%
	I do not remember	6	5.2%
What is the time period between the birth of the child and the wound being sutured? (N=116)	Less than an hour after the cut	107	92.2%
	An hour or more after the cut	9	7.8%
Did they put anesthesia before cutting the perineum? (N=116)	Yes	52	44.8%
	No	39	33.6%
	I don't know	25	21.6%
If you have ever had your perineum cut, what were the reasons? (N=116)	Facilitating and accelerating childbirth	65	56.0%
	Routine procedure	39	33.6%
	To assist in delivery	37	31.9%
	To avoid perineal tear	36	31.0%
	Pregnant for the first time	34	29.3%
	A large baby	26	22.4%
	Previous history of perineal tear	9	7.8%
	Other	8	6.9%
	Maternal fatigue from pushing during birth	7	6.0%
	Fetal distress (weak fetal heartbeat)	4	3.4%
	Upon the patient’s request	3	2.6%
	Breech baby	2	1.7%
	Need to use obstetric forceps or vacuum.	0	0.0%
	I don't know	0	0.0%
Are there any complications after cutting the perineum? (N=116)	Yes	72	62.1%
	No	44	27.9%
If yes, what type of complications occurred (N=116)	Perineal pain	39	33.6%
	Dyspareunia	31	26.7%
	Disintegration of the suture	25	21.6%
	Infection	21	18.1%
	Increased possibility of cuts and incisions in the next birth	19	16.4%
	Incontinence	12	10.3%
	Extension of the tear to the cervix	5	4.3%
	Extension of the tear to the third or fourth degree (meaning it reaches the anus)	5	4.3%

The association between various sociodemographic characteristics and the knowledge levels of episiotomy and wound care among 252 participants was also explored. It indicates significant differences in knowledge scores across different age groups (p=0.014), with participants aged over 45 exhibiting the highest mean knowledge score of 15.2 (SD=3.1, median=15.0, IQR=14.0-17.0), followed by those aged 35-44 (mean=14.1, SD=3.1, median=14.0, IQR=12.0-16.0). Marital status also showcased a noteworthy association (p=0.036), with married participants having a higher mean knowledge score of 14.3 (SD=3.1, median=14.0, IQR=12.0-16.0) compared to others. The number of births was significantly linked to knowledge scores (p=0.008), where participants with five or more births displayed the highest mean score of 14.6 (SD=2.8, median=14.0, IQR=13.0-16.0). Conversely, no significant associations were found between knowledge scores and nationality (p=0.474) or education level (p=0.190). This analysis highlights distinct disparities in knowledge regarding episiotomy and wound care concerning age, marital status, and the number of childbirths among the participants (Table 5).

**Table 5:** Association of Sociodemographic Characteristics With Knowledge of Episiotomy and Wound Care ^K^Independent-samples Kruskal-Wallis test. ^U^Independent-samples Mann-Whitney U test. *p<0.05, significant. The data has been represented as mean±SD and median.

		Knowledge Score (0-25)				
		Mean	SD	Median	IQR	P value^K,U^
Age group	15-24	13.5	2.9	13.0	11.0-16.0	0.014*
	25-34	13.3	3.0	13.0	11.0-15.0	
	35-44	14.1	3.1	14.0	12.0-16.0	
	More than 45	15.2	3.1	15.0	14.0-17.0	
Nationality	Non-Saudi	13.0	2.7	13.0	11.0-14.0	0.474
	Saudi	13.9	3.1	14.0	12.0-16.0	
Education	High school and below	13.1	2.9	13.5	11.0-16.0	0.190
	University and above	14.0	3.1	14.0	12.0-16.0	
Marital status	Divorced	13.6	1.4	13.5	12.5-14.5	0.036*
	Married	14.3	3.1	14.0	12.0-16.0	
	Single	13.1	3.0	13.0	11.0-15.0	
	Widow	12.0	4.2	12.0	9.0-15.0	
Number of births	1-4	14.2	3.1	14.0	12.0-16.0	0.008*
	5 or more	14.6	2.8	14.0	13.0-16.0	
	None	13.1	3.0	13.0	11.0-15.0	

## Discussion

This study aimed to determine the perceptions and knowledge of women in the Qassim region, Saudi Arabia, regarding episiotomy. A good chunk of participants (62.7%)(N=402) showed that they know about episiotomy, indicating that people in the community are interested in this topic. A big number (94.0%) out of N=402 correctly identified episiotomy as a surgical cutting with scissors, showing that they have a basic understanding to make informed decisions. Looking at other studies, Zaidan et al. (2018) found a similar high awareness rate (63.6%) [14], suggesting that women worldwide care about understanding childbirth-related procedures [15]. The consistent recognition of episiotomy as a surgical procedure shows that it is a common concern for women globally [16].

A notable finding is that 82.5% of participants (N=402) knew that not all women need episiotomies. This lines up with a study by Zaidan et al. (2018), where 38% of participants understood that the procedure is selective [14]. These findings stress the importance of making it clear that episiotomy is not necessary for everyone, something that should be part of education efforts. But there is a difference in knowing the need for anesthesia before episiotomy (48.8%)(N=402). Carroli et al. (2009) also reported a similar awareness rate (46.6%) in their study [17]. This emphasizes that there is a need for specific education in this area.

A positive finding is that 90.5% of participants believed that there are ways to avoid perineal cutting, in line with the findings of Bonet et al. (2017) [18]. This supports the idea of empowering women with preventive measures, like knowing how to push during childbirth (69.4%) and doing exercise (54.4%). Talking about post-cutting care, the focus on antibiotics (61.5%), analgesia (52.8%), and laxatives (48.8%) (N=402) matches with what Mohamed et al. (2021) found [19]. This shows a good overall understanding of taking care of oneself after episiotomy.

Looking into post-episiotomy wound care knowledge, participants showed a good understanding, similar to Mohamed et al. (2012) findings [20]. Most agreed on hygiene practices, changing sanitary pads (97.2%), and keeping the wound clean and dry (98.8%), showing a global understanding of crucial care practices [21]. However, the differing opinions on certain practices, like using herbs, resemble the findings of Hadizadeh-Talasaz et al. (2022), who reported varying cultural beliefs [22]. This stresses the need for educational efforts that respect diverse beliefs and practices.

The experiences of those who had episiotomies align with O'Kelly et al. (2017), emphasizing the need for better communication during consent, timely suturing, and post-procedure support [23]. Complications reported by Maphanga et al. (2021) emphasize the importance of addressing and lessening complications through tailored care plans [24].

This study has practical implications for healthcare practices in the Qassim region, Saudi Arabia. Educational initiatives are crucial to address specific knowledge gaps identified, particularly the need for anesthesia awareness and diverse beliefs about post-episiotomy care. Healthcare providers can leverage these insights to develop educational materials that are culturally sensitive, using clear and accessible language to enhance awareness and misconceptions. By understanding the unique perspectives of women in the Qassim region, healthcare professionals can foster an environment that empowers women to make informed decisions about their care, contributing to improved maternal health outcomes.

Future research should explore deeper into the cultural factors influencing women’s perceptions of episiotomy in the Qassim region. This includes an exploration of how cultural norms and traditions shape perspectives, informing more targeted and effective interventions. Longitudinal studies tracking the impact of educational initiatives on women’s knowledge and decision-making can provide valuable insights into the sustained effectiveness of interventions aimed at improving awareness and reducing unnecessary episiotomies. Additionally, examining the experiences and perspectives of healthcare providers in the region regarding episiotomy practices will offer a dual perspective, enriching our understanding of the factors influencing decision-making and care. This holistic approach to research can contribute to comprehensive strategies that align healthcare practices, ultimately enhancing maternal care and childbirth experiences.

It is essential to acknowledge the limitations of this study. The research design, likely cross-sectional, may restrict the ability to establish causality or capture changes over time. The reliance on self-reported data introduces the possibility of recall bias, as participants may not accurately remember or disclose certain information about their experiences or knowledge. The sample size of 402 participants, while providing useful data, may not fully represent the diverse perspectives within the Qassim community, limiting the generalizability of findings. Additionally, the study predominantly focuses on women’s perspectives, and the absence of healthcare provider perspectives may overlook crucial insights into episiotomy practices and decision-making. Despite these limitations, the study serves as a foundational exploration of episiotomy awareness in the Qassim region, offering a basis for future research and targeted interventions.

## Conclusions

The study reveals a notable awareness among participants, with a majority demonstrating a solid understanding of the procedure, its indications, and post-procedure care. It identified specific knowledge gaps, such as the need for anesthesia awareness and divergent beliefs about post-episiotomy care practices. The study highlights avenues for future research, encouraging a deeper exploration of cultural factors influencing perceptions and a longitudinal examination of the sustained impact of educational initiatives on women's knowledge and decision-making.

Enhancing intrapartum decision-making and communication between patients and obstetricians is of utmost importance. To achieve this, we have developed a comprehensive action plan that includes implementing evidence-based educational programs for obstetricians and pregnant women, developing user-friendly decision support tools, providing communication skills training to obstetricians, creating patient decision aids for informed choices, establishing a feedback mechanism for continuous improvement, and organizing volunteer campaigns with trained individuals who offer support and act as advocates. By implementing these initiatives, we aim to enhance women's knowledge, promote shared decision-making, and improve communication during childbirth.
